# Preparation of Porous Scaffolds from Silk Fibroin Extracted from the Silk Gland of *Bombyx mori* (*B. mori*)

**DOI:** 10.3390/ijms13067762

**Published:** 2012-06-21

**Authors:** Mingying Yang, Yajun Shuai, Wen He, Sijia Min, Liangjun Zhu

**Affiliations:** Institute of Applied Bioresource Research, College of Animal Science, Zhejiang University, Yuhangtang Road 866, Hangzhou 310058, China; E-Mails: 21117047@zju.edu.cn (Y.S.); 21017041@zju.edu.cn (W.H.); minsj@zju.edu.cn (S.M.); ljzhu@zju.edu.cn (L.Z.)

**Keywords:** *B. mori* fibroin, silk gland, porous scaffolds, compressive strength, cell adhesion and growth activity, ALP activity

## Abstract

In order to use a simple and ecofriendly method to prepare porous silk scaffolds, aqueous silk fibroin solution (ASF) was extracted from silk gland of 7-day-old fifth instar larvae of *Bombyx mori* (*B. mori*). SDS-page analysis indicated that the obtained fibroin had a molecular weight higher than 200 kDa. The fabrication of porous scaffolds from ASF was achieved by using the freeze-drying method. The pore of porous scaffolds is homogenous and tends to become smaller with an increase in the concentration of ASF. Conversely, the porosity is decreased. The porous scaffolds show impressive compressive strength which can be as high as 6.9 ± 0.4 MPa. Furthermore, ASF has high cell adhesion and growth activity. It also exhibits high ALP activity. This implies that porous scaffolds prepared from ASF have biocompatibility. Therefore, the porous scaffolds prepared in this study have potential application in tissue engineering due to the impressive compressive strength and biocompatibility.

## 1. Introduction

The silk spun from domestic silkworm *Bombyx mori* (*B. mori*) has been used commercially for biomedical sutures for several decades, and in textile production for at least 2500 years. The silk from *B. mori* consists primarily of two protein components: Fibroin is the structural protein of silk fibers and sericins are the water-soluble glue-like proteins that bind the fibroin fibers together [1]. It has been proven that *B. mori* silk fibroin has superior characteristics, including impressive mechanical properties, biocompatibility, moisture and oxygen permeability, promoting proliferation of human keratinocytes and human skin fibroblasts [2–5]. In addition, *B. mori* silk fibroin can be processed into forms of film, gel, porous material and non-woven mats [6–12]. Therefore, the silk fibroin has been used as an important set of material options in the fields of controlled release, biomaterials, and scaffolds for tissue engineering [13–15].

Recently, three-dimensional (3D) porous scaffolds based on *B. mori* silk fibroin have been increasingly proposed for the field of tissue engineering because, not only the porous scaffolds have enough mechanical stability to support cell adhesion and expansion, and degrade at a rate comparable with new tissue growth, but also porosity of the scaffold is critical to provide sufficient opportunity for cell migration and expansion [16–20]. It has been reported that porous silk scaffolds are useful for healing critical size femur defects in rats [21]. The scaffolds seeded with chondrocytes can support cartilage tissue engineering. It has been found that 3D highly porous silk scaffolds are suitable for osteogenesis and chondrogenesis of human bone marrow stem cells (hMSCs) [18]. The features of 3D porous silk scaffolds are related to cartilage tissue engineering from hMSCs [22].

It is well known that silk fibroin is synthesized and stored in the silk gland with a high concentration of aqueous solution. However, after formation of silk fiber, fibroin is insoluble in most solvents, including water, dilute acid and alkali and resists digestion from most proteolytic enzymes. A number of solvents including lithium bromide and lithium thiocyanate are used to regenerate *B. mori* silk fibroin from cocoon [23,24]. Porous scaffolds are usually obtained from silk fibroin solution by using organic solvents such as Hexafluoroisopropanol (HFIP) [25,26]. However, these solvents are harsh, difficult to recycle, and environmentally unfriendly. In order to avoid application of organic solvents, the Kaplan group developed porous scaffolds based on an aqueous regenerated silk fibroin solution [27,28]. Recently, Kunda *et al*. [29,30] reported that it is possible to extract the silk fibroin from the silk gland of a mature fifth instar non-mulberry Indian tropical tasar silkworm, *Antheraea mylitta* (*A. mylitta*), in large scale to prepare scaffolds. Extraction of silk fibroin from silk gland not only avoids the use of organic solvents and harsh chemicals but can also avoid chemical degradation of the fibroin, suggesting a simple and ecofriendly method to prepare silk-based scaffolds. Both *B. mori* and *A. mylitta* are silkworms that produce cocoon silk, therefore, it seems feasible to extract the fibroin from the silk gland of *B. mori* to prepare scaffolds.

In this study we attempted to prepare porous scaffolds by isolating fibroin from silk gland of 7-day-old fifth instar larvae of *B. mori*. The molecular weight and amino acid composition were confirmed. The morphology of porous scaffolds was observed with SEM, and porosity was measured by using mercury intrusion porosimeter. The compressive strength of porous scaffolds was tested. The structure was characterized with FT-IR. The biocompatibility, including cell adhesion and cell growth activity, and ALP activity was assayed.

## 2. Results and Discussion

### 2.1. Identification of ASF

**Figure 1:**
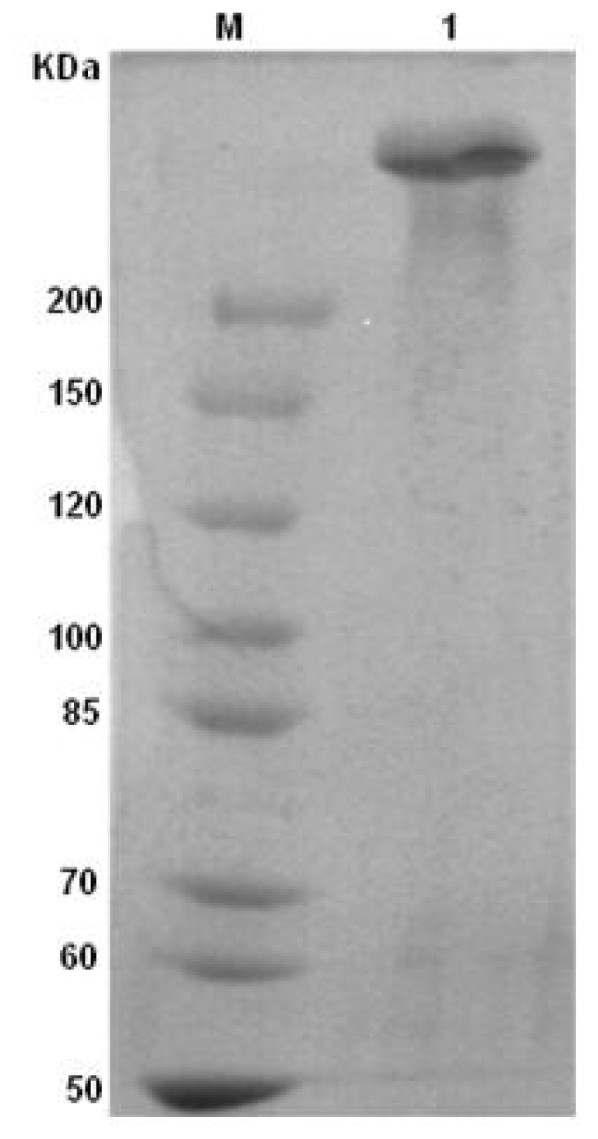
SDS-page analysis of aqueous silk fibroin (ASF). M: Marker; lane 1: ASF.

The molecular weight of ASF was estimated according to SDS-page electrophoresis. Figure 1 shows the SDS-page gel stained with Coomassie Blue R-250 for ASF. As shown in Figure 1, the mark appeared one band higher than that located at 200 kDa against molecular weight mark, meaning that the ASF molecular weight was higher than 200 kDa. Compared to the molecular weight of undegraded native fibroin (about 350 kDa) and generated fibroin prepared from CaCl_2_/ethanol/H_2_O solvent system (100–20 KDa) [31,32], ASF might not degrade when extracted from silk gland. An amino acid analysis of ASF was also performed. As seen in Table 1, the content of amino acid composition is similar to the results reported by Shimura *et al.* [33]. This is also consistent with the SDS-page analysis, further implying that the ASF extracted from the silk gland remains intact.

### 2.2. Morphology of Porous Scaffolds

**Figure 2:**
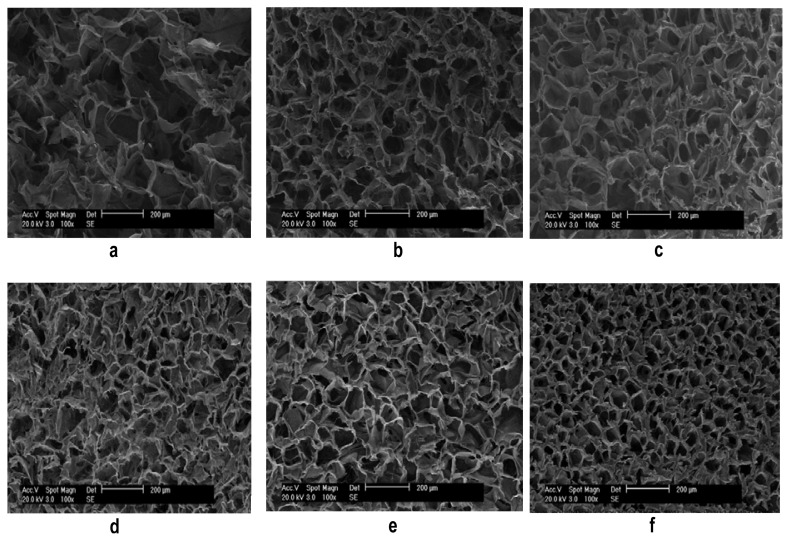
Scanning electron micrograph of porous scaffolds prepared from ASF with a concentration of: (**a**) 2 wt%; (**b**) 4 wt%; (**c**) 6 wt%; (**d**), (**e**), (**f**) correspond to (**a**), (**b**) and (**c**) respectively after being treated with ethanol.

The porous scaffolds were successfully prepared from ASF with a concentration of 2 wt%, 4 wt% and 6 wt%, respectively. These three kinds of scaffolds were treated with ethanol. Figure 2 shows scanning electron micrographs of the cross section of the porous scaffolds before and after ethanol treatment. The morphology is uniform, and the pore size distribution is homogeneous in all the scaffolds. The pore size appears largest when the scaffolds were prepared from fibroin with a concentration of 2 wt% (Figure 2a). On increasing the concentration to 4 wt% and 6 wt%, the pore size decreases (Figure 2b,c). Comparing Figure 2a to 2d, and Figure 2c to 2f, respectively, it can be seen that the pore size decreases significantly after the scaffolds are treated by ethanol.

In addition, the porosity, pore diameter and apparent density of porous scaffolds were measured using mercury intrusion porosimeter. Table 2 lists the porosity, pore diameter and apparent density of these scaffolds. As can be seen in Table 2, the characteristics of the scaffolds depend on preparation conditions. The scaffolds prepared with a concentration of 6 wt% and treated by ethanol have lowest porosity (91.4%), smallest pore diameter (17,736 nm) and highest apparent density (1.8 mg/L), suggesting that the morphology, porosity and pore diameter, *etc*. of scaffolds can be controlled by changing the concentration of the fibroin or the post treatment.

### 2.3. The Mechanical Properties of Porous Scaffolds

**Figure 3:**
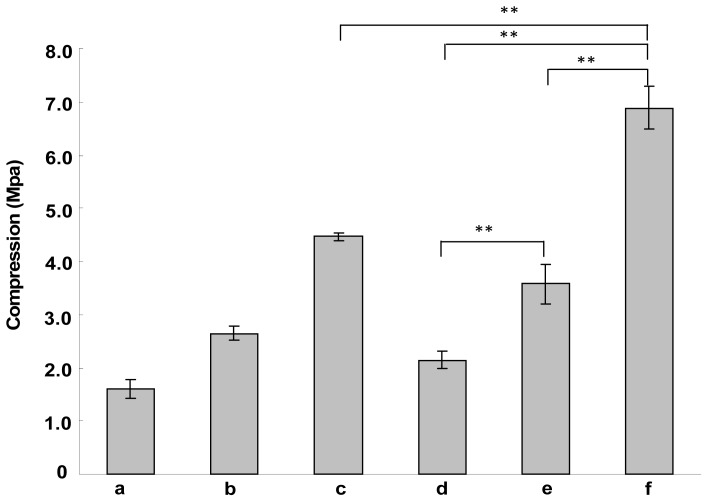
The mechanical properties of porous scaffolds prepared from ASF with a concentration of (**a**) 2 wt%; (**b**) 4 wt%; (**c**) 6 wt%; (**d**), (**e**), (**f**) corresponding to (**a**), (**b**) and (**c**) treated by ethanol, respectively. Each value represents the mean value ± SD. ** *p* < 0.01.

The mechanical properties of porous scaffolds prepared from ASF with different concentrations were measured. Figure 3 indicates the compressive strength of these scaffolds. As shown in Figure 3, the compressive strength increases by increasing the concentration of ASF. The compressive strength increased from 1.6 ± 0.2 Mpa through 2.6 ± 0.1 Mpa to 4.5 ± 0.1 Mpa when the concentration of ASF was increased from 2 wt% through 4 wt% to 6 wt% (Figure 3a–c). After ethanol treatment, the compressive strength of these scaffolds is increased to 2.1 ± 0.2 Mpa, 3.6 ± 0.4 Mpa and 6.9 ± 0.4 Mpa, respectively. Ethanol treatment can improve the compressive strength significantly (*p* < 0.01). The porous scaffolds prepared from ASF with a concentration of 6 wt% and treated by ethanol have highest compressive strength, significantly higher (*p* < 0.01) than that of porous scaffolds that were prepared from ASF with concentrations of 2 wt% and 4 wt%. It has been reported that the mechanical properties of scaffolds is dependent on the composition and microstructure, *etc*., for example, uniform pore distribution and low porosity can improve mechanical properties [34,35]. Therefore, this high compressive strength might be attributed to the high molecular weight of ASF, the uniform pore distribution and the low porosity of porous scaffolds.

### 2.4. Structure Characterization of Porous Scaffolds

**Figure 4:**
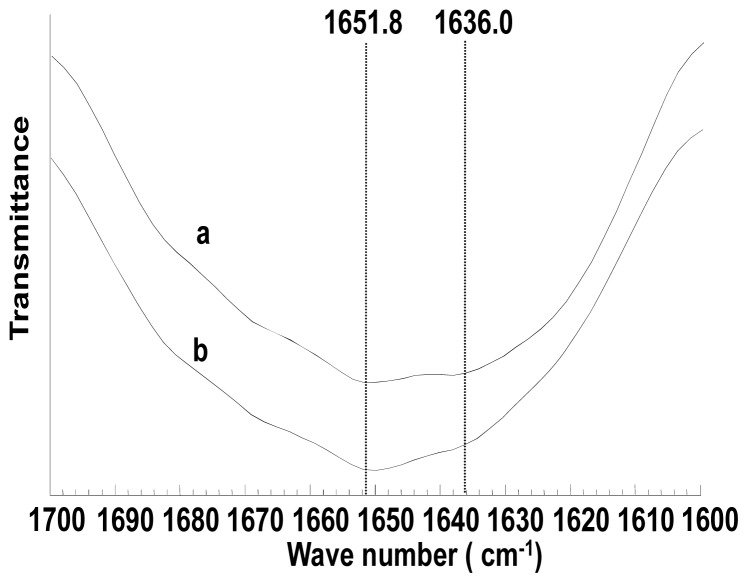
FTIR spectra of porous scaffolds prepared from ASF (**a**) without ethanol treatment (**b**) treated by ethanol.

FTIR was used to confirm secondary structure of porous scaffolds prepared from ASF. Figure 4 shows the amide I region of the FT-IR spectra of porous scaffolds before and after ethanol treatment. Amide I absorption is the most useful for estimating protein secondary structures, because it arises predominantly from C=O stretching vibration. As shown in Figure 4, the appearance of amide I band at 1651.8 cm^−1^ and 1636.0 cm^−1^ indicated that scaffolds adopt random coil conformation with a large number of β-sheet conformation. No difference was observed on the secondary structure of porous scaffolds before and after ethanol treatment. Therefore, the difference in the compressive strength of porous scaffolds before and after ethanol treatment might be due to the tertiary/quaternary structure and the morphology such as porosity, apparent density and pore size, *etc*.

### 2.5. Cell Adhesion and Growth Activity of ASF

**Figure 5:**
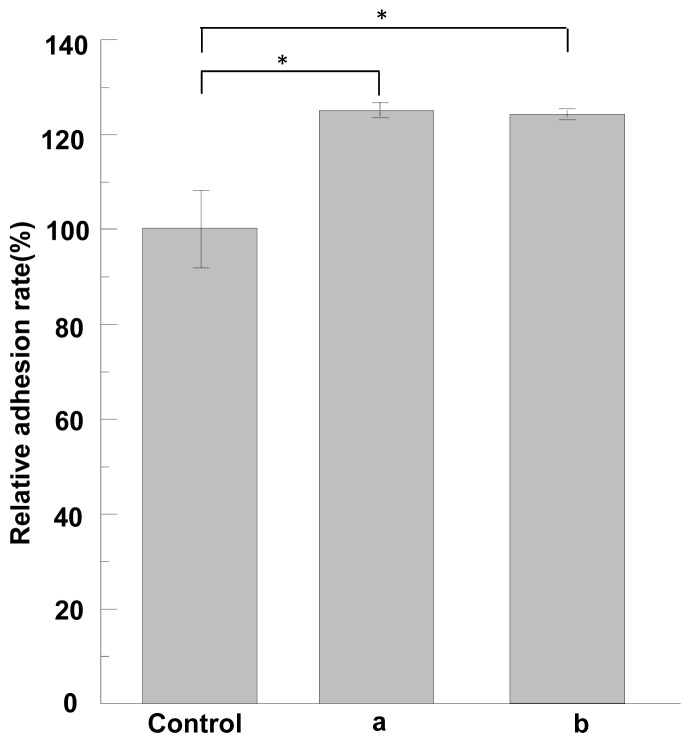
The cell adhesion assay of ASF (**a**) induced by formic acid, (**b**) induced by TFA. Non-treatment plate was used as control. Each value represents the mean value ± SD. * *p* < 0.05.

**Figure 6:**
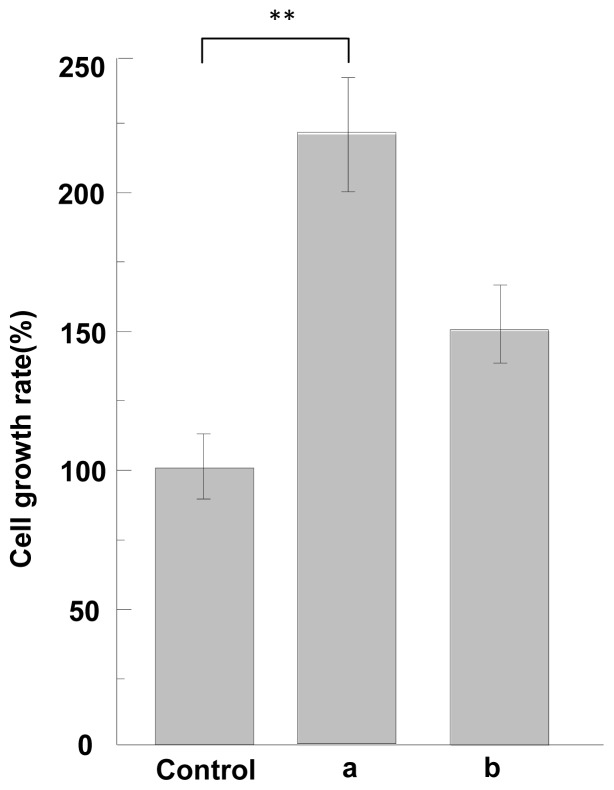
The cell growth assay of ASF (**a**) induced by formic acid, (**b**) induced by TFA. Non-treatment plate was used as control. Each value represents the mean value ± SD. ** *p* < 0.01.

The cell adhesion and growth activity of ASF were measured by using a non-treatment plate as control. The cell adhesion activity of ASF in response to MG-63 cells is shown, relative to the non-treatment plate (100%), in Figure 5. Here the results were analyzed according to the statistical treatment, *t*-test. As compared with the control, ASF induced by formic acid or TFA has higher cell adhesion (*p* < 0.05) as shown in Figure 5. Figure 6 shows the cell growth activities of ASF compared to non-treatment plate. ASF induced by formic acid or TFA shows higher cell growth activities than control. Especially, ASF induced by formic acid exhibits cell growth activities significantly higher than that of control (*p* < 0.01). Therefore, it can be concluded that ASF extracted from silk gland has high cell adhesion and growth activity, indicating that cells can attach well on the scaffold when the ASF is formed into scaffolds.

### 2.6. ALP Activity Assay of ASF

**Figure 7:**
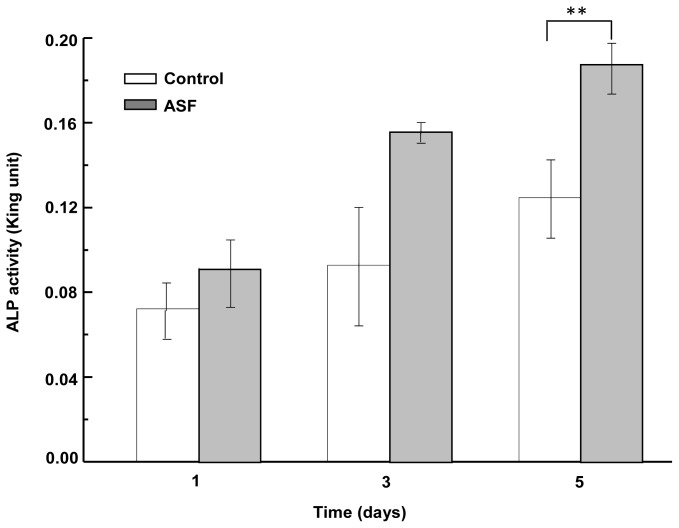
ALP activity assay of MG-63 cells grown on ASF for 1 days, 3 days and 5 days. Non-treatment plate was used as control. Each value represents the mean value ± SD. ** *p* < 0.01.

ALP increasing activity is an early marker of osteoblast phenotype and differentiation. In order to investigate the differentiation of MG-63 on ASF, ALP activity was measured by culturing the cells on ASF for 1 day, 3 days and 5 days. Figure 7 shows ALP activity after cells were cultured on ASF for 1 day, 3 days and 5 days, respectively. As shown in Figure 7, ALP activity of ASF was increased with increase in cultivation day. After 5d of cultivation, ALP activity of ASF was significantly higher than that of control (*p* < 0.01). This implied that the scaffolds prepared from ASF are favorable for cell differentiation.

## 3. Materials and Methods

### 3.1. Materials

The 7-day-old fifth instar larvae of *B. mori* were provided by Huzhou Academy of Sericulture, China. MG-63 cells were purchased from Shanghai Institute of Life Sciences Research, Chinese Academy of Sciences. All chemicals were of analytical grade and purchased from Huipu Chemical Agents Co. Ltd., China.

### 3.2. Extraction of ASF from the Silk Gland of *B. mori*

**Figure 8:**
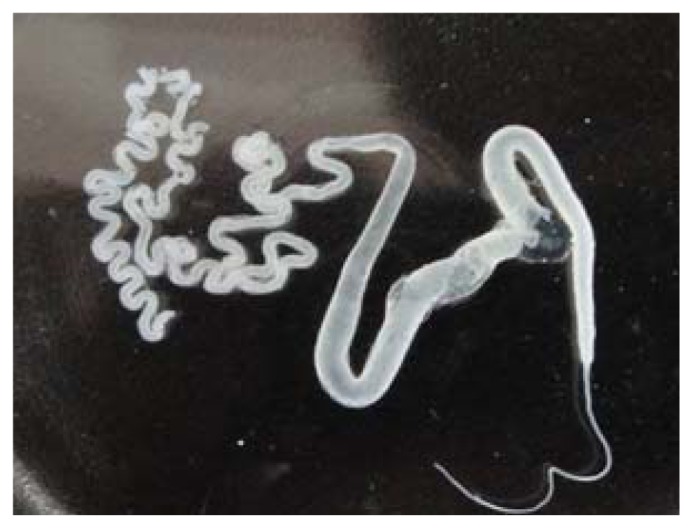
The silk gland of the 7-day-old fifth instar larvae of *B. mori* silkworms.

The silk gland as shown by Figure 8 was pulled out with forceps from the abdominal side of the 7-day-old fifth instar larvae of *B. mori*. According to the previously reported method [36], the middle part of silkglands were taken out and washed twice in ice deionized water. After removal of epithelium, the silkglands were immersed in distilled water to remove most of soluble sericin protein. After that, the residues were collected into a beaker containing deionized water and shaken softly for 1 h at 0 °C. After centrifuged, the aqueous silk fibroin solution (ASF) was obtained, which was used to prepare porous scaffolds.

### 3.3. Amino Acid Composition Analysis

Molecular weight of ASF was determined according to SDS-page electrophoresis. The gel was stained with Coomassie brilliant blue R250 after electrophoresis. In addition, the amino acid composition of ASF was analyzed. ASF was air-dried and hydrolyzed in 6 N HCl for 48 h at 110 °C. After removal of HCl, the concentration of the residues was diluted to be 0.1% by using 0.2 N sodium citrate (pH 2.2). The amino acid analysis was carried out using Hitachi L8900 Amino Acid Analyzer.

### 3.4. Preparation of Porous Scaffolds

The concentration of ASF was controlled at 2 wt%, 4 wt% and 6 wt%, respectively. The ASF solution with various concentrations was added to 24 well microplates. After placed at room temperature overnight, ASF solution was aggregated and turned into gel. The porous scaffolds were formed by freezing these gels at −20 °C for 24 h followed by lyophilization. Post treatment was performed by soaking porous scaffolds into ethanol for 24 h followed by air dried. Ethanol treatment was expected to change the mechanical properties of porous scaffolds according to the previous study [37].

### 3.5. Scanning Electron Microscopy

The morphology of porous scaffolds was observed by scanning electron microscopy (XL30-E, PHILIPS) at a voltage of 20 kV. The samples were sputtered coated with gold. Samples were examined under vacuum, and images were recorded digitally.

### 3.6. Porosity of Porous Scaffolds

The porosity, pore diameter and apparent density of porous scaffolds were determined by using mercury intrusion porosimeter (AutoPore IV 9510, Micromeritics, USA) Samples were subjected to a pressure cycle starting at approximately 0.5 psia, increasing to 60,000 psia in predefined steps to give pore size/pore volume information.

### 3.7. Mechanical Property Test

The mechanical properties of porous scaffolds were measured using mechanical testing machine (AGS-J, Shimadzu, Japan) at room temperature with a 50 N load cell. The porous scaffold measuring 12 mm in diameter and 10 mm in height were used for the tests. The compressive strength was determined (*n* = 4) by drawing a line parallel to the 1% strain: the intersection point of the line with the stress–strain curve gave the compressive strength of the scaffolds.

### 3.8. Fourier Transformed Infrared Spectroscopy

Infrared spectra were collected using a FTIR spectrometer (SHIMADZU 8400s). The porous scaffolds were pressed into discs with KBr with ratio of 1:20. The measurements were performed with a scanning frequency of 100 times. Infrared spectra were taken using a Perkin-Elmer system 2000 Fourier transform IR spectrometer.

### 3.9. Cell Adhesion Assay

MG-63 cells were used for the cell adhesion assay. In order to compare the cell adhesion and growth activity of ASF that is in different structural state, ASF was dissolved in formic acid and Trifluoroacetic acid (TFA), respectively. Formic acid can induce *B. mori* silk fibroin to adopt β-sheet conformation while TFA can induce random coil and β-sheet structure [38]. The solution with concentration of 1 mg/mL was prepared by dissolving ASF in formic acid and TFA, respectively, and was added to 24-well microplates, 200 μL/well. The plates were desiccated under vacuum for 2 h after drying at room temperature for 12 h. The wells were washed with physiological saline twice, and then, saturated with 0.2% BSA for 2 h. Before adding 200 μL DMEM medium, the wells were washed twice with physiological saline. The plates were kept in a 37 °C incubator for 1 h. Cultured cells were washed with PBS, detached with 0.25% trypsin (Invitrogen, USA), washed, and suspended again in DMEM medium. This cell suspension with volume of 200 μL was added to wells with total number of 1.0 × 10^5^ cells/mL, and incubated for 60 min at 37 °C, 5% CO_2_. The number of suspension cells was counted by inserting Scepter™ handheld automated cell counting (Millipore, USA) into cell suspension. The final number of cells was obtained by subtracting suspension cells from total cells.

### 3.10. Cell Growth Assay

Cell growth assay was also performed by using MG-63 cells. Cell growth plates were prepared as same as cell adhesion assay, until the plates were desiccated under vacuum condition. After washing with physiological saline twice, 200 μL DMEM medium was added and the plates were kept in a 37 °C incubator for 1 h. Cultured cells were washed with PBS, detached with 0.25% trypsin, washed and re-suspended to approximately 1.0 × 10^5^ cells/mL in DMEM medium. This cell suspension was added to wells which were kept in the incubator, so that the number of cells was 2.0 × 10^4^ cells a well, and incubated for 3 days at 37 °C, 5% CO_2_. After removal of the supernatants, the wells were washed with PBS twice and detached by adding 0.25% trypsin. The cells were harvested, and the number of cells was counted using Scepter™ handheld automated cell counting (Millipore, USA).

### 3.11. ALP Activity Assay

ASF with concentration of 1 mg/mL was added to 24-well plates, 200 μL/well. After drying at room temperature for 12 h, the plates were desiccated under vacuum for 2 h, and then incubated in DMEM overnight at room temperature. After the DMEM was removed, the MG-63 cells were seeded at a concentration of 5.0 × 10^3^ cells/well on scaffold incubated in DMEM medium containing 10% FBS. The cells were incubated at 37 °C in 5% CO_2_ for 1 day, 3 days and 5 days, and the medium was replaced every 2 days. The ALP activity in the cell lysates was measured based on the conversion of colorless *p*-nitrophenyl phosphate into colored *p*-nitrophenol (JianCheng Biotech., China). The color intensity (OD_sample_) was measured at 520 nm using the UV–Vis spectrophotometer (DU530, Beckman Coulter, USA). The amount of ALP was quantified by the comparison with the intensity of phenol standard solution (OD_standard_) according to formulation:

$$\text{ALP activity }(\text{king}\;\text{unit}/100\;\text{mL})=\frac{{\text{OD}}_{\text{sample}}}{{\text{OD}}_{\text{standard}}}\times 0.005\;\text{mg}\times \frac{100\;\text{mL}}{0.05\;\text{mL}}$$

Here, 1 mg phenol formed in the reaction of 100 mL serum with substrate at 37 °C for 15 min was considered as 1 king unit.

### 3.12. Statistical Analysis

Experiments including mechanical property test, cell adhesion and growth assay and ALP activity assay were repeated four times. Results were presented as mean values ± standard deviation (SD). Significant differences were evaluated by one-way level variance analysis. The statistical significance in two tailed *t*-test and p value were calculated.

## 4. Conclusions

In this study, in order to prepare porous silk scaffolds by using a simple and ecofriendly method, aqueous silk fibroin solution (ASF) was extracted from silk gland of 7-day-old fifth instar larvae of *B. mori*. The porous scaffolds were prepared from ASF in concentrations of 2 wt%, 4 wt% to 6 wt%. The morphology, porosity and mechanical properties of porous scaffolds are dependent on the concentration of ASF and post-treatment. The compressive strength of porous scaffolds can be as high as 6.9 ± 0.4 MPa. The structure of porous scaffolds was mainly random coil conformation with a β-sheet structure according to FTIR analysis. The cell adhesion and growth assay combined with ALP activity test proved that porous scaffolds prepared from ASF have biocompatibility. Therefore, it has been proven that extraction of fibroin from silk gland of *B. mori* to prepare scaffolds is an effective, ecofriendly method. Furthermore, the porous scaffolds prepared in this study show promise for potential application in tissue engineering.

## Figures and Tables

**Table 1:** The amino acid composition analysis of ASF.

Amino acid	ASF	Reference [33]	Amino acid	ASF	Reference [33]
Ala	30.0	30.0	Val	2.3	2.5
Gly	43.8	42.9	Leu	0.6	0.6
Tyr	2.5	4.8	Ile	0.6	0.6
Ser	11.0	12.2	Phe	1.6	0.7
Asp	2.1	1.9	Pro	0.8	0.5
Arg	0.6	0.5	Thr	1.0	0.9
His	0.2	0.2	Met	0	0.1
Glu	2.5	1.4	Cys	0	0
Lys	0.4	0.4			

**Table 2:** The porosity, average pore diameter and apparent density of porous scaffold prepared from ASF measured by mercury intrusion porosimeter, and compressive strength (*n* = 4, mean ± SD).

Concentration (wt%)	Ethanol treatment	Porosity (%)	Average pore diameter (nm)	Apparent density (g/mL)	Compression (Mpa)
2	No	90.9	24,679	1.1	1.6 ± 0.2
4	No	88.9	19,524	1.2	2.6 ± 0.1
6	No	85.1	7,736	1.3	4.5 ± 0.1
2	Yes	91.4	22,848	1.1	2.1 ± 0.2
4	Yes	88.4	19,786	1.4	3.6 ± 0.4
6	Yes	82.8	14,466	1.8	6.9 ± 0.4
